# Peer Reviewed Publication Skills Matter for Academicians

**DOI:** 10.30699/ijp.2020.129608.2426

**Published:** 2020-10-10

**Authors:** Payam Behzadi

**Affiliations:** 1 *Department* of Microbiology, College of Basic Sciences, Shahr-e-Qods Branch, Islamic Azad University, Tehran, Iran

**Dear Editor**

On July 21 (Sunday) and 23 (Tuesday) 2019, I had an opportunity to hold another workshop titled “How to write a scientific paper?” for Ph.D. students in Microbiology Department, College of Basic Sciences, Islamic Azad University, Shahr-e-Qods Branch, Tehran, Iran. I received brilliant feedback from the participants encouraging me to publish this letter to the editor. 

The difficult work of writing is an art like rasping and grinding a rough diamond to have a polished and smooth diamond (1) or like making a film (2). Publishing peer-reviewed papers is a criterion for evaluating academic professionals and is an effective means of forming an academic career and resume (3). 

As we know since the ancient era, the published texts are used as public official means to communicate with diverse people and populations. According to the latest archeological studies, the first handwriting belongs to 10400 years ago discovered in Baluchestan Province, the southeast of Iran (4).

The primitive means of scripts have evolved and now we have high-tech tools to publish different types of scripts as softcopies and hardcopies, as well as traditional and online publications (4, 5). English as an international language may connect all people around the world. Therefore, publishing scholarly papers in English language journals promotes the visibility and citation of the papers (5, 6). 

A strong scientific paper needs a clear roadmap, a well-designed study, and an up-to-date quality proposal. Blurry hypothesis, poor-designed study, biases, inappropriate sample size (e.g., very small and limited populations or samples), and wrong or insufficient statistical analyses are the most common reasons for the rejection of papers (6-8).

I as a non-native English-speaking author and reviewer believe that the non-native English-speaking authors should think, imagine, and write their manuscripts in English. Many young non-native English-speaking authors write their manuscripts in their native language and then translate into English. This is very harmful because it makes your manuscript of poor quality in academic English language, results in more probability of rejection (7). 

**Fig. 1.**
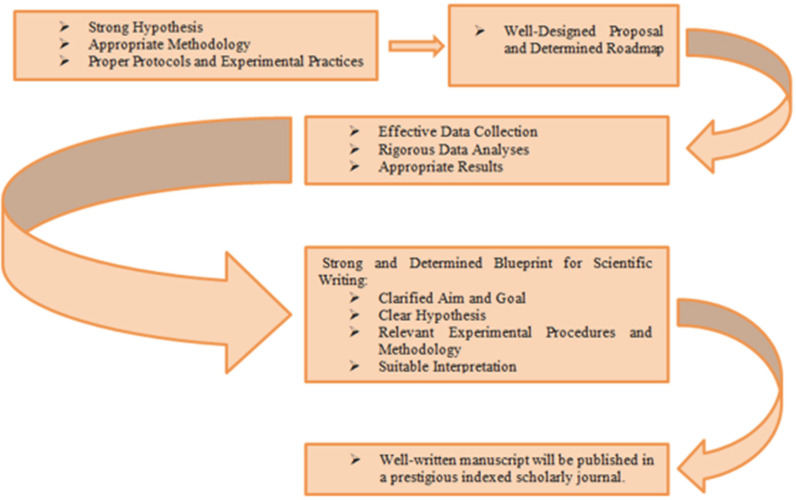
Successful process of publishing a strong manuscript in a peer-reviewed journal

Consequently, in this challenge, the authors should prepare a well-planned manuscript with a strong and clear hypothesis (it goes back to the proposal) and relevant aims. Moreover, proper statistical analyses and calculations, correspondent methodology, appropriate and precise conclusion, sharp and clear figures, and well-designed and self-explanatory tables augment the opportunity of a manuscript for acceptance (Figure 1) (4, 6, 7, 9, 10).
